# Determination of Drying Characteristics and Physicochemical Properties of Mint (*Mentha spicata* L.) Leaves Dried in Refractance Window

**DOI:** 10.3390/foods13182867

**Published:** 2024-09-10

**Authors:** Mohammad Kaveh, Shahin Zomorodi, Szymanek Mariusz, Agata Dziwulska-Hunek

**Affiliations:** 1 Agricultural Engineering Research Department, West Azerbaijan Agricultural and Natural Resources Research and Education Center, AREEO, Urmia 5716963963, Iran; sirwankaweh@gmail.com; 2 Department of Agricultural, Forest and Transport Machinery, University of Life Sciences in Lublin, Głęboka, 28, 20-612 Lublin, Poland; 3 Department of Biophysics, University of Life Sciences in Lublin, 20-612 Lublin, Poland; agata.dziwulska-hunek@up.lublin.pl

**Keywords:** artificial neural network (ANN), drying, energy, essential oil

## Abstract

Drying is one of the most common and effective techniques for preserving the quantitative and qualitative characteristics of medicinal plants in the post-harvest phase. Therefore, in this research, the effect of the new refractance window (RW) technology on the kinetics, thermodynamics, greenhouse gasses, color indices, bioactive properties, and percentage of mint leaf essential oil was investigated in five different water temperatures in the form of a completely randomized design. This process was modeled by the methods of mathematical models and artificial neural networks (ANNs) with inputs (drying time and water temperature) and an output (moisture ratio). The results showed that with the increase in temperature, the rate of moisture removal from the samples increased and as a result, the drying time, specific energy consumption, CO_2_, NO_x_, enthalpy, and entropy decreased significantly (*p* < 0.05). In addition, the drying water temperature had a significant effect on the rehydration ratio, color indices, bioactive properties, and essential oil percentage of the samples (*p* < 0.05). The highest value of rehydration ratio was obtained at 80 °C. By increasing temperature, the main color indices such as b*, a*, L*, and Chroma decreased significantly compared to the control (*p* < 0.05). However, with the increase in temperature, the overall color changes (ΔE) and L* first had a decreasing trend and then an increasing trend, and this trend was the opposite for the rest of the indicators. The application of drying water temperature from 50 to 70 °C increased antioxidant, phenol content, and flavonoid content, and higher drying temperatures led to a significant decrease in these parameters (*p* < 0.05). On the other hand, the efficiency of the essential oil of the samples was in the range of 0.82 to 2.01%, and the highest value was obtained at the water temperature of 80 °C. Based on the analysis performed on the modeled data, a perceptron artificial neural network with 2-15-14-1 structure with explanation coefficient (0.9999) and mean square error (8.77 × 10^−7^) performs better than the mathematical methods for predicting the moisture ratio of mint leaves.

## 1. Introduction

In recent decades, the desire to use plant materials as a potential source of bioactive compounds has increased with the aim of focusing on meeting the ever-increasing demand of the pharmaceutical, food, and cosmetic industries, as well as creating added value in agricultural and pharmaceutical products [1]. Mint with the scientific name (*Mentha spicata* L.) is one of the most important medicinal plants used in Iran and the world which contains essential oil. Mint essential oil is used in the pharmaceutical industry for the purpose of deodorizing the mouth and in the production of toothpaste and mouthwash [2]. Mint leaves, flowers, and stems are traditionally used as herbal teas and spices in many foods to add flavor and aroma [3]. Mint was originally used as a medicinal plant to treat stomach pain and chest pain [4]. Dried mint leaves are widely used as a seasoning and have a high economic value, but drying should be carried out in a way to produces a product with maximum aroma and taste.

Drying, as one of the oldest methods of food preservation, includes mass and heat transfer phenomena simultaneously [5]. Drying has advantages such as increasing the shelf life; reducing the volume, weight, and packaging of the product; reducing the costs of transportation and storage; and the possibility of obtaining fruits, vegetables, medicinal plants, and seasonal foods throughout the year and with the excellent quality [6].

In previous studies, mint leaves have been dried by different methods, including fluidized bed [7]; infrared individually, and combined with different methods [1,8,9]; continuous-dehumidified air [4]; heat pump [10]; hot air (HA) [11]; electrohydrodynamics [6]; freezing [5,12]; and microwave individually and combined with other methods [3,13]. Each of these mentioned methods has limitations. In HA and fluidized bed dryers, the product is in direct contact with HA, which causes changes in the physicochemical properties, and they also have low energy efficiency [14]. In this regard, Mokhtarikhah et al. [15] reported that the drying time of spearmint leaves using a HA dryer is very long. On the other hand, the results of some studies have shown that drying chrysanthemums using the HA method, compared to other methods, causes an increase in energy consumption and a decrease in bioactive properties [16]. Freeze dryers are approximately three to seven times more expensive than HA dryers [17]. Microwave, infrared, and electrohydrodynamic methods are laborious, costly, and require expensive equipment and capital. In addition to the significant effect of the drying process on the shelf life of products, the results of some studies have also shown that the method used for drying has a significant effect on performance, maintaining bioactive properties, improving quality properties, and increasing the yield of essential medicinal plants [18,19].

Considering the importance of drying medicinal plants, this operation should preserve the qualitative, bioactive, and efficiency characteristics of the essential oil. Also, energy consumption is another significant factor in the process of drying herbal plants [20]. Drying with the RW method is one of the new and advanced technologies used for drying medicinal plants, which has emerged to deal with the problem of long time, low quality, and high energy in different dryers [21].

RW drying is a novel and non-thermal technique (fourth generation of dryers) in the pharmaceutical, nutritional, cosmetic, and health industries and is used for various products that are puree [22,23], powder [24], pulp [25], leather [26], paste [27], and fruit slices [21,28]. In addition, this technique is used for heat-sensitive products such as fruits [29], vegetables [30], and medicinal plants [20]. In this method, the plant is flatly spread on a Mylar plate (polyester film), which is placed in hot water (as a heating medium) [31].

The transfer of heat and removal of moisture by circulating hot water is carried out by conduction and radiation [29]. Due to the characteristics of water in the rapid transfer of thermal energy, the moisture in the product evaporates quickly and as a result, thermal balance is established [32]. The benefits of the RW drying method compared to other drying methods incorporate the demand for low operational tasks [33]; high evaporation and mass transfer rates [34]; shorter drying time [35]; the proper shelf life of dry products [30]; the uniform drying of products [29]; the preservation of aroma, taste, color, and vitamins [21]; the improvement of the bioactive properties of dry products [26]; lower water activity [14]; good stability during storage [25]; lower costs (about 30–50%) [20]; lower energy consumption [36]; and affordability [22]. as Along with preserving the quality of dried products, thermal efficiency and high energy efficiency of RW dryer systems, which are usually in the range of 55 to 72% and 28 to 38%, respectively, are the other advantages of this technique [32,37]. 

In this regard, Zamani et al. [20] showed that the drying method with RW compared to infrared, shade, HA, and combined infrared-RW methods for drying Dracocephalum kotschyi, in addition to maintaining the qualitative properties, has recorded maximum bioactive properties and minimum microbial contamination properties of the plant. The results of the study Dadhaneeya et al. [28] showed that RW drying maintains heat-sensitive compounds to a greater extent with better product appearance and in less time compared to HA and vacuum methods.

With the progress in computer technologies, the control and measurement of the drying process can be properly managed by monitoring the control of the drying conditions (moisture in the drying process) and providing operational information about the performance of the drying system [38].

Artificial neural network (ANN) is one of the most common methods for modeling processes. Among the reasons for the high popularity of ANN compared to other classical modeling methods are the ease of working with it, the great ability of ANN in forecasting, the possibility of reducing or increasing input and output variables, and the ability to use it to predict more than two variables in output [39]. By choosing the best model that describes the drying kinetics, it is possible to study the process of product drying changes during the process and provide better industrial dryers according to the type of product [40]. ANN method has been widely used in order to guide and skillfully solve complex nonlinear problems, model food industry processes, and predict the desired parameters in the design and development of systems [38].

Zalpouri et al. [39] observed that an ANN model predicts the value of moisture ratio (MR) for coriander with RW dryer with a high R^2^ value compared to the mathematical model. The research conducted by Selvakumarasamy et al. [41] used ANNs to accurately analyze and model the parameters of moisture ratio and moisture content of Insulin plant leaves in the context of an HA drying system. Similarly, the work of Mahesh et al. [40] and Kalsi et al. [42] showed the application of ANN in accurately depicting the moisture content and moisture ratio of drying Annona muricata and Stevia rebaudiana, respectively, using convective technology. Their emphasis was on more application and use of the wide potential of artificial neural networks in the agricultural sector and beyond.

Therefore, the review of the reported studies illustrates that using the RW method is a promising and practical drying method due to its low energy consumption and low processing cost to produce dried products with high quality and nutritional value and better consumer acceptance. Also, considering the importance of medicinal and aromatic plants (such as mint) and the necessity of their correct processing, it seems that using this method can increase the quality of the final product in addition to decreasing the time and specific energy consumption (SEC) of the drying process. In spite of the reports of numerous research about the successful use of RW dryers in drying different products, so far there has been no study related to its effect on the kinetics, thermodynamic properties, greenhouse gasses, essential oil, and bioactive indices of medicinal and aromatic plants such as mint and there is a significant gap in the existing knowledge related to the analysis of this segment. Therefore, in this research, mint leaves were dried in an RW dryer and the effect of water temperature on the drying time, process energy consumption, thermodynamic properties, greenhouse gasses, rehydration ratio, color characteristics, preservation of active biological components, and also the yield of essential oil extractable from the dried samples was investigated. In addition, six mathematical models and artificial neural networks are used in predicting the MR with the number of neurons, algorithms, and different activation functions for modeling.

## 2. Materials and Methods

Healthy and undamaged mint plants (*Mentha spicata* L.) were carefully harvested by hand from a field in Urmia, West Azerbaijan, before the flowering stage, at a height of 5 cm from the ground. The scientific name of a fresh plant sample was confirmed at the West Azerbaijan Medicinal Plants Research Center. In order to preserve the quality of the plant, until the beginning of the experiments, the harvested samples were placed in layers and inside wet cotton cloths in a refrigerator at a temperature of 4 ± 1 °C. In order to determine the initial moisture content of mint leaves, four 10 g samples of leaves were carefully weighed and placed in an oven (Memmert, UFB500, Schwabach, Germany) at a temperature of 105 °C for 4 h [43]. The initial humidity of the mint leaves in these tests was 80 ± 0.5% (w.b%). 

### 2.1. Refractance Window Dryer

**Figure 1:**
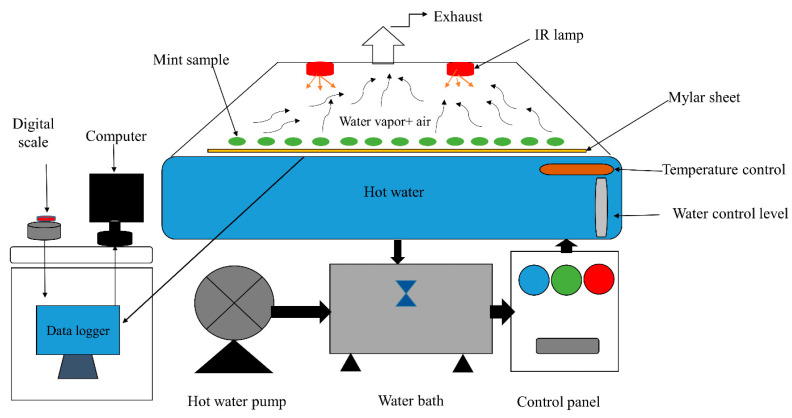
A schematic diagram of the refractance window drying system.

To dry mint, an RW dryer was used on a laboratory scale (Food Industry Engineering Department, Urmia Agricultural Research Center, West Azarbaijan, Iran-schematic, Figure 1). The RW dryer was made of a Mylar plate (transparent plastic film) with a thickness of 0.26 mm and an area of 50 × 200 cm^2^) as a plastic membrane. In this dryer, the Mylar film was placed on the source of hot water at temperatures of 50, 60, 70, 80, and 90 °C, and the plant samples were distributed on the surface of the Mylar film. The RW dryer consists of a 100 L static thermal bath made of steel. In order to remove the vapors resulting from the moisture evaporation of the drying mints, an industrial electric fan was used in the dryer. Two electric heaters were used to supply hot water to the system, and a centrifugal water pump with a speed of 2900 rpm was used to circulate the hot water. The parts of the device were installed on a steel skeleton with a length of 2.5 m and the electronic control equipment of water temperature, motor control, pump, and ventilators were installed in a control panel. All the experimental works were carried out in laboratory conditions (24 °C and 30% relative humidity). The mint sample was placed on the Mylar plate, uniformly and in a single layer, and their weight loss was measured and recorded at specified time intervals, every 5 min, and then the trend curve of the moisture changes in the drying product with time was drawn.

### 2.2. Drying Kinetics

In most research, the drying kinetics is reported based on the moisture ratio index (MR). Equation (1) was used to calculate the MR of the mint leaves during the drying process [7,44]:

(1) $$MR=\frac{M_{t}-M_{e}}{M_{o}-M_{e}}$$

### 2.3. Modeling

For the mathematical modeling of sample drying, MR was used, whose equations are derived from the relationship between the changes in the moisture values and drying time. For this purpose, the six mathematical models of drying shown in Table 1 were used. In order to find the best model and obtain their coefficients, using the nonlinear regression method and MATLAB R2019a software, the above models were fitted to the data obtained from the different stages of the dryer.

### 2.4. Diffusion Coefficient and Activation Energy

Fick’s second law of percolation (Equation (2)) is widely used to describe the drying process in the range of decreasing moisture rates for agricultural products [41]:

(2) $$\frac{\partial x}{\partial t}=D_{eff}\frac{\partial^{2}x}{\partial x^{2}}$$

The penetration equation for thin blade coordinates has been solved by considering assumptions [47] and for long drying times, it is expressed as a logarithmic Equation (3) [48].

(3) $$\ln(MR)=\ln(\frac{8}{\pi^{2}})-\ln\left(\frac{\pi^{2}}{4L^{2}}D_{eff}.t\right)$$

The D_eff_ is obtained from the slope of Equation (3). From Equation (3), by drawing the graph of Ln(MR) against time, a straight line with a slope was obtained, which can be calculated using Equation (4) (related to the slope of the line) [2]:

(4) $$K_{1}=\left(\frac{D_{eff}\pi^{2}}{4L^{2}}\right)$$

Based on the Arrhenius relationship, the dependence of D_eff_ on temperature can be expressed [49]. This can be seen in the following link:

(5) $$D_{eff}=D_{o}\exp\left(\frac{E_{a}}{RT}\right)$$

The value of E_a_ can be calculated from the slope of the graph of ln(D_eff_) against 1/(T + 273.15).

### 2.5. Thermodynamic Properties

Equations (6)–(8) were used to analyze the thermodynamic behavior (enthalpy, entropy, and Gibbs free energy) of mint under different drying temperatures by the RW method considering the global gas constant (R = 8.314 kJ/mol·K) [50].

(6) $$\Delta H=E_{a}-RT$$

(7) $$\Delta S=R\left[\ln(D_{0})-\ln\left(\frac{K_{b}}{h_{p}}\right)-\ln(T)\right]$$

(8) ΔG=ΔH−TΔS

### 2.6. Specific Energy Consumption

Equation (9) shows the SEC for drying mint, which was proposed by Zalpouri et al. [39] for the RW method. First, the electrical energy consumed during the drying process was measured using a digital energy meter (BENETECH GM86, Shenzhen, Guangdong, China).

(9) $$SEC(kWh/kg)=\frac{\;\mathrm{Total}\;\mathrm{energy}\;\mathrm{supllied}\;\mathrm{in}\;\mathrm{drying}\;(\mathrm{kWh})}{\;\mathrm{Quantity}\;\mathrm{of}\;\mathrm{moisture}\;\mathrm{removed}\;\mathrm{during}\;\mathrm{drying}\;(\mathrm{kg})}$$

### 2.7. CO_2_ and NOx

To calculate the emission of CO_2_ greenhouse gasses produced for drying mint in the RW method, it is assumed that 1 kilowatt hour of energy from a combined-cycle power plant with fossil fuel (gas oil) has been used. In addition, on the basis of the report of Darvishi et al. [51] and Nazari et al. [52], the CO_2_ and NO_x_ coefficients used for the combined-cycle power plant and fossil fuel (oil and gas) are 622 g/kWh and 3.78 g/kWh, respectively. Therefore, based on Equation (10) provided by Motevali et al. [53], the amount of CO_2_ was calculated.

(10) $$CO_{2}=SEC_{real}\times EF\times \left(1-\frac{ER}{100}\right)$$

(11) $$SEC_{real}=\left(\frac{SEC}{0.871}\right)$$

### 2.8. Color

To check the amount of changes (∆E) in the color characteristics of food during the drying process, color indicators are used. These indices include brightness or L* from black to white, redness or a* from green to red, and yellowness or b* from blue to yellow [41]. After extracting the values of the a*, L*, and b* indices by a CR 400 model machine, Konica Minolta, Tokyo, Japan, before and after drying, the overall color changes (Equation (12)) and Chroma index (Equation (13)) for all the samples were calculated and evaluated [11].

(12) $$\Delta E=\sqrt{{\left(L_{0}^{*}-L^{*}\right)}^{2}+{\left(a_{0}^{*}-a^{*}\right)}^{2}+{\left(b_{0}^{*}-b^{*}\right)}^{2}}$$

(13) $$C=\sqrt{{\left(a^{*}\right)}^{2}+{\left(b^{*}\right)}^{2}}$$

### 2.9. Rehydration Ratio

Experiments to measure RR by immersing 4 g of the dried mint leaf samples in distilled water at 50 °C for 30, 60, 90, 120, and 150 min were performed. The RR in each time period was calculated using Equation (14) [38].

(14) $$RR=\frac{W_{r}}{W_{d}}$$

### 2.10. Measurement of the Total Phenol Content (TPC)

The amount of TPC compounds in the mint was evaluated using the Folin–Ciocalteu colorimetric method [54]. In this method, the TPC compounds were calculated in terms of mg GAE/100 g dw. The procedure was that 20 μL of the prepared extract was mixed with 1.16 mL of distilled water and 100 μL of Folin reagent was added to the above solution. After 5 min, 300 μL of 20% sodium carbonate solution was added to the solution and the samples were kept in a bain-marie at 40 °C for 30 min after stirring with a tube stirrer. Then, the absorbance of the samples was read with a spectrophotometer at a wavelength of 760 nm.

### 2.11. Measurement of the Total Flavonoid Content (TFC)

A total of 5 mL of the extract was added to 2 mL of distilled water and 0.15 mL of NaNO_2_ (5%) solution. Exactly 10 min later, 0.15 mL of AlCl_3_ solution (10%) was added to the mixture. After 10 min, 2 mL of potassium acetate solution (5%) was added to the mixture and immediately taken to 5 mL with distilled water. The absorbance of the samples was measured by a spectrophotometer after 30 min of storage at a wavelength of 510 nm and the result was reported as mg QE/100 g dw of the sample [9]. In order to draw the standard curve of quercetin, 5 mg of quercetin was brought up to volume with 80% ethanol in a 10 mL flask and different concentrations of quercetin were prepared, and the steps explained to measure the absorption of the extract were repeated for different concentrations of quercetin.

### 2.12. Measurement of Antioxidant Activity (AA) by DPPH Method

DPPH is a lipophilic radical that has maximum absorption at 517 nm wavelength. First, 1 mL of the methanolic solution of 1 mM DPPH was mixed with 3 mL of the solution of production extracts and mixed vigorously. The resulting mixture was kept for 30 min at room temperature in the dark and finally, their absorbance was read at the wavelength of 517 nm. The AA was calculated in terms of the relative percentage of DPPH according to Equation (15) [29].

(15) $$AA(\%)=(1-\frac{A_{sample}}{A_{Control}})\times 100$$

### 2.13. Extraction of Essential Oil (EO) by Distillation with Water

Distillation with water and a Clevenger device were used to extract the EO of the samples. A total of 35 g of the dried mint samples in each treatment were transferred into a balloon with a capacity of 1500 mL. Then, 1000 mL of distilled water was added to it and the process of extracting the EO was performed for 3 h [55].

### 2.14. Artificial Neural Networks

**Figure 2:**
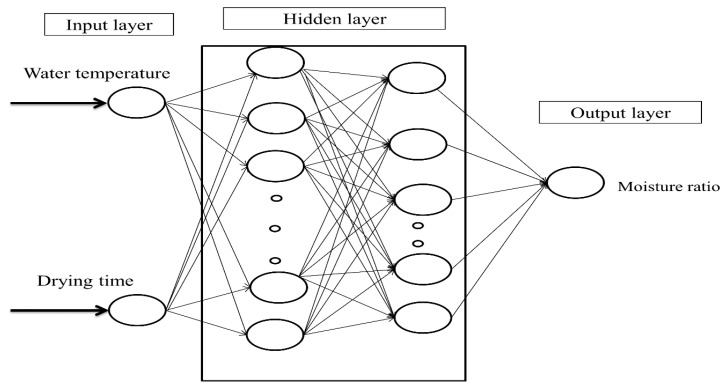
Schematic diagram of ANNs with two input and two hidden layers for moisture ratio.

ANN is a notion to process information that is influenced by the biological nervous system and processes information similar to the brain. The main factor of this notion is the new structure of the information processing system. This system contains numerous highly interconnected processing elements that act together to figure out a problem. ANNs, like humans, learn through example. An ANN is configured to accomplish a specific task, including identifying patterns and classifying information, throughout a process of learning. In biological systems, learning is connected with adaptations in synaptic connections between nerves. It is a calculation method which relies on the interconnected connection of several processing units. The network incorporates an arbitrary number of neurons, which, by recognizing the inherent relationships between the data, provide a mapping between the input space (input layer) and the desired space (output layer). The modeling of the mint drying process using the RW method to predict MR using the ANN method was carried out by three- or four-layer feedforward perceptron neural networks (Figure 2). This network consists of an input layer, one or more hidden layers, and an output layer. The maximum number of repetitions required in the network learning process was considered 1200.

In this study, the drying time and drying water temperatures were considered as the input parameters, and MR as the target (output) parameter. In predicting the MR, 70% of the data were randomly used as training data, and 30% of them were used as test (15%) and validation (15%) data. Also, the Levenberg–Marquardt algorithm, activation function (linear, sigmoid, and hyperbolic tangent), and changing the type of hidden layer neuron number (2–15) in perceptron neural network were used. The performance of the network was evaluated using the mean square error (MSE-Equation (16)) and correlation coefficient (R2-Equation (17)) in order to evaluate the obtained results [42].

(16) $$MSE=\frac{1}{N}\sum_{i=1}^{N}{\left(MR_{pre,i}-MR_{\exp,i}\right)}^{2}$$

(17) $$R^{2}=1-\frac{\sum_{i=1}^{N}{\left[MR_{\exp,i}-MR_{pre,i}\right]}^{2}}{\sum_{k=1}^{N}{\left[\frac{\sum_{k=1}^{n}MR_{pre,i}}{N}-MR_{pre,i}\right]}^{2}}$$

### 2.15. Statistical Analysis

The analysis of variance (ANOVA) and Duncan’s multi-range test were used to compare the average data. The confidence level used for the statistical analysis was considered 95% and all the analyses were performed by the SPSS version 21 software.

## 3. Results and Discussion

### 3.1. Moisture Ratio

**Figure 3:**
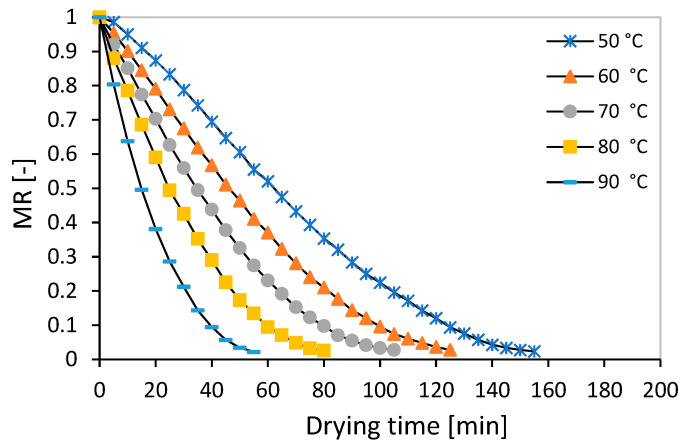
Moisture ratio vs. drying time at different water temperature levels.

The drying curves at different water temperatures are given in Figure 3. As can be seen in Figure 3, at the beginning of the drying process, the rate of moisture loss of the mint leaves is high; gradually and with the passage of time, the product’s moisture level decreases and the rate of moisture loss naturally decreases. The mint leaves lose most of their moisture in the early stages of drying. As a result, it is clear that the slope of the drying curve increases with the increase in temperature so it has the highest slope at the drying water temperature of 90 °C. The possible reason for this can be the reduction in pores inside the tissue of the mint leaves and their shrinking due to shriveling. For every 10 °C increase in water temperature, the drying time is reduced by 20 to 30 min. These results were similar to the results of the drying kinetics of thin layers of *Curcuma longa* L. in the RW method [56]. From the research that was performed on the trend in moisture increase with time, it can be found that the mint leaves lost most of their moisture during the period of decreasing speed and this period is the most important period of the drying of this product. So, the dominant mechanism of moisture transfer during the drying of this product is the mechanism of water vapor diffusion. The results of the analysis of variance (ANOVA) show that the drying air temperature had a significant effect on the drying time (*p* < 0.01—Table 2). According to the average comparison table (Table 3), by increasing the water temperature for drying mint leaves, the evaporation potential increases and the temperature has a direct effect on the transfer of moisture from inside the product to the outside and causes a rapid decrease in the moisture of mint leaves. Its drying time is reduced. A similar result of this research regarding the direct effect of temperature on the speed of the thin layer drying process has been reported for some medicinal plants such as coriander [39] and Dracocephalum kotschyi [20].

### 3.2. Values of D_eff_ and Activation Energy

Table 3 shows the effect of different temperatures on D_eff_. Temperature has an effect on the D_eff_, and with increasing temperature, the D_eff_ has an increasing trend (Table 2, *p* < 0.01). The lowest and highest values of D_eff_ were calculated as 2.48 × 10^−8^ and 7.28 × 10^−8^ m^2^/s, respectively. The creation of molecular movement and surface suction is the reason for the increase in D_eff_ with increasing temperature [49]. Also, the increase in temperature increases the enthalpy of the incoming air, and the increase in enthalpy increases the rate of mass and heat transfer, which increases the D_eff_. The values of D_eff_ and E_a_ for agricultural products and food are reported by researchers in the range of 10^−12^ to 10^−8^ m^2^/s and 12.7 to 110 kJ/mol, respectively [46,57]. The D_eff_ for drying mint in a HA dryer in the temperature range of 40 to 60 °C in the range of 5.09 × 10^−9^ m^2^/s to 1.73 × 10^−8^ m^2^/s has been reported [11]. Also, in another study, the D_eff_ for mint in the forced convection method was reported from 6.12 × 10^−9^ to 1.19 × 10^−8^ m^2^/s [2]. The results of this research are consistent with the reports of other researchers. The E_a_ for drying mint at different water temperatures was 25.75 kJ/mol. Researchers reported the E_a_ of mint leaves as 32.25 kJ/mol in the solar dryer [58], 52.89 kJ/mol in the HA method [11], 60.82 to 65.59 kJ/mol in the fluidized bed technique [59], and 79.07 to 67.44 kJ/mol in the freeze method [5].

### 3.3. Thermodynamic Properties

The average values of enthalpy changes (ΔH), Gibbs free energy (∆G), and entropy change (∆S) for drying mint in the RW method are given in Table 4. ΔH is the energy difference between the reactant and the activated complex during the drying process [50], ΔG is the basic measure for the spontaneity of the chemical reaction [46], and ∆S is the change factor of the order of molecules in a system [60]. The value of ΔH was between 22.76 and 23.10 kJ/mol and its value increased with increasing water temperature. Furthermore, positive ΔH values indicate an endothermic reaction (androgenic reaction) and indicate that the reaction requires energy [50]. The ΔH values of water temperature of 90 °C showed a significant decrease of 1.4% compared to the drying water temperature of 50 °C. These findings show that lower water temperature requires more energy than higher water temperature for the moisture evaporation process during mint drying. Santos et al. [61] and Cunha et al. [56] reported a similar process for yam and *Curcuma longa* L. in the RW method, respectively.

As can be seen from Table 4, the change in entropy (∆S) is negative (−0.1175 to −0.1077 kJ/mol °K) and the trend in its changes is similar to enthalpy; that is, it decreased with increasing temperature. This decrease in entropy is due to the decrease in water content, which leads to a more restricted movement of water molecules, which is attributed to a decrease in available sites. Santos et al. [60] recorded such a trend for mango and stated that this entropy behavior indicates a process with exothermic transformations. Almeida et al. [62] also reported that entropy decreases as a result of increased diffusion when drying Achachairu, providing less molecular heterogeneity.

The increase in temperature increased the Gibbs free energy (∆G) from 57.90 to 65.43 kJ/mol (Table 4). The positive values of ∆G indicate the non-spontaneous nature of the mint drying process in the RW method. de Vilela Silva et al. [50] and Gomes et al. [63] showed that during the drying of mangosteen peel and Jambu leaves, respectively, the ∆G has a positive tendency and increases with the drying temperature. Considering that this parameter represents the most energy released in a process, therefore, it requires an influx of energy from the surrounding atmosphere to weaken the water content through the phase transition from liquid to vapor. 

### 3.4. Specific Energy Consumption

The average values related to SEC at different temperatures are shown in Table 3. The average SEC of the process of drying mint leaves in the RW dryer at the temperatures of 50, 60, 70, 80, and 90 °C was calculated using the laboratory data and Equation (9) and 5.51, 4.91, 4.21, 3.53, and 2.81 kWh/kg were obtained, respectively. Increasing the temperature from 40 to 90 °C caused a 49% decrease in the SEC. The values obtained in this research with the results reported by other researchers such as Rajoriya et al. [26] for banana leather 2.80 kWh/kg, Baeghbali et al. [33] for pomegranate juice from 4.31 kWh, and Zalpouri et al. [39] for coriander is comparable from 7.88 to 9.10 kWh/kg in an RW dryer. Based on the obtained laboratory results and statistical analysis (Table 2 and Table 3), the increase in air temperature caused a significant decrease in SEC for the process of drying mint leaves (*p* < 0.01). Increasing moisture evaporation and reducing process time due to the increase in thermal gradient at higher temperatures is the main reason for reducing the SEC of the process [37]. Observations similar to this finding have been reported by different researchers such as Ye et al. [1] for mint and Xing et al. [64] for Gentiana macrophylla.

### 3.5. CO_2_ and NOx

The effects of drying water temperature on CO_2_ and NOx emissions are given in Table 2. The results indicate that in an RW combined dryer, the effect of dryer water temperature on CO_2_ and NO_x_ values is significant (*p* < 0.05). According to Table 3, CO_2_ emission varies between 1.94 and 3.80 kg/kg water. The highest and lowest levels of NO_x_ were 0.023 kg/kg water and 0.011 kg/kg water, respectively. Increasing the temperature from 50 to 90 °C caused a decrease of 48.9 and 52.1% of CO_2_ and NO_x_. Increasing the temperature of the drying water, due to the faster evaporation of the product’s free water, reduces the drying time, SEC, and as a result, reduces the emission of greenhouse gasses (CO_2_ and NO_x_). Seyfi et al. [37] dried Aloe vera using HA and RW and observed that increased air temperature reduced CO_2_ and NO_x_ emissions. Kaveh et al. [65] reported that the emission of CO_2_ and NO_x_ ranged from 59 to 166 kg/kg water and 0.36 to 1.01 kg/kg water, respectively, during the HA drying of pear in a fuel-fired combined-cycle power plant.

### 3.6. Color

Color is one of the most important quality characteristics of dried agricultural products and it changes during drying and long-term storage due to some chemical and biochemical reactions. Table 5 shows the effect of different water temperatures on the color characteristics of mint leaves dried with RW. The average values of the L* (lightness), a* (redness) and b* (yellowness), ∆E, and Chroma indices after drying were obtained between 32.75 and 39.03, −9.13 and −7.18, 21.28 and 26.93, 9.96 and 13.00, and 22.46 and 28.43, respectively (Table 6). In this study, the value of a (redness–greenness index) was used as an index to describe the degradation of pigments in the mint leaves; its increase indicates more redness and its decrease indicates the greenness of the sample. This index includes positive and negative values, and the more negative its value, the more the color of the samples tends to be green. In this experiment, the value of this index in the control sample was the highest (−10.80); in addition, the lowest value (−7.18) was related to the water temperature of 50 °C, and with the increase in the water temperature, its value significantly had an increasing trend at first and then a decreasing trend (*p* < 0.01). Nalawade et al. [19], in the study they conducted on the color change in dried mint, observed that the value of a* significantly decreases with increasing temperature from 30 to 50 °C, and its negative value decreases which indicated the decrease in the green color of the dried mint samples.

The +b index indicates increased yellowness and −b indicates increased blueness. As can be seen in Table 6, with the increase in the water temperature from 50 to 70 °C, there was a significant increasing trend, but from 70 to 90 °C, this index decreased significantly and the yellow color of the samples became less. The highest value (27.87) of this index was seen in the control treatment, which had a significant difference with all the treatments (*p* < 0.01). A similar trend was reported in the study by Ertekin and Heybeli [8]. The lowest value was related to the water temperatures of 60 °C and 90 °C (*p* < 0.01). 

The color parameter L*, which is related to the darkening of the tissue of fruits, medicinal plants, and vegetables, is due to enzymatic and non-enzymatic reactions. The value of the darkness–lightness index after drying is between 32.75 and 39.03, and its high value indicates that it is lighter and its decrease indicates the darkness of the samples. The highest value of L* was obtained in the fresh sample with a value of 42.89 and the lowest value was obtained at a water temperature of 50 °C with a value of 32.75, which is due to the long drying process. As can be seen, the value of this index increased significantly with the increase in water temperature up to 70 °C and then decreased significantly with the increase in water temperature up to 90 °C (*p* < 0.01). Therefore, mint leaves dried at lower temperatures were significantly darker than leaves dried at other temperatures (*p* < 0.01). In all the drying treatments, this trait was significantly different from the control sample and was lower than in the fresh sample. Kripanand et al. [66], Beigi, [11], and Ertekin and Heybeli [8] obtained similar results in the study of mint drying with different methods. In the water temperature of 80 °C, the results were almost the same as in the water temperature of 70 °C and there was a slight decrease. But at the highest water temperature (90 °C), this index showed a further decrease.

The ΔE index indicates color changes during sample drying, and its large value indicates a greater difference from the control sample in terms of color. As can be seen in Table 6, the changes in ΔE are in the range of 9.96 to 13.00, and the highest value was obtained at the water temperature of 50 °C. Tan et al. [67] stated that long drying time due to low process temperature can change the color of products and a browning reaction occurs. The lowest amount of color changes was obtained at 70 °C and 80 °C water temperature, and there was no statistically significant difference (*p* < 0.01). An increase in the temperature first caused a decrease in color changes and then an increase. Therefore, color changes increased at 90 °C water temperature compared to 70 °C and 80 °C because high temperature causes sudden color change due to the non-enzymatic Maillard reactions during drying. 

Chroma determines the degree of purity of a color. The results showed that the highest value of the Chroma index (28.43) with a significant difference was related to the dried sample at 70 °C water temperature (*p* < 0.01). Its lowest (22.46) was related to 50 °C temperature treatment. In addition, its increase trend was observed with increasing temperature from 50 to 70 °C. But there was no significant difference in the 60 and 90 °C treatments (*p* < 0.01). The fresh samples showed better Chroma values than the dried samples and this indicates that the fresh samples are purer and brighter than the dried samples.

### 3.7. Rehydration Ratio

**Figure 4:**
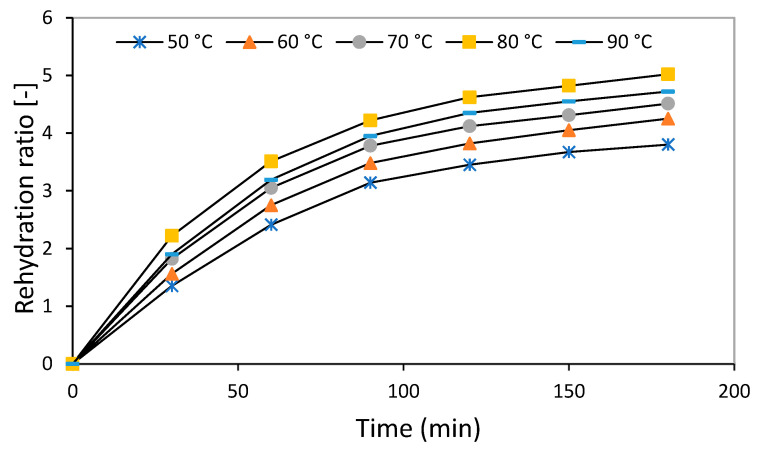
The rehydration ratio of the dried mint leaf varieties conducted at 50 °C.

In general, water reabsorption is a complex phenomenon that is influenced by various factors, such as the method and conditions of performance, as well as the inherent characteristics of the product. It should be noted that due to the changes that occur in the structure and physical and chemical characteristics of biological products during the drying process, water absorption by dry products with a cellular structure (biological products) is much more complicated. The water reabsorption capacity of dried products is one of the most important features and is one of the quality indicators of these products. The average values of the RR of the dried mint samples at the temperatures of 50, 60, 70, 80, and 90 °C were obtained as 3.8, 4.25, 4.51, 5.02, and 4.72, respectively. For example, Beigi [11] reported the RR of dried mint leaves in the range of 5.25 to 5.64. On the other hand, the results of the analysis of variance (ANOVA) showed a significant effect of drying temperature on mint rehydration (*p* < 0.01—Table 7). Figure 4 shows the behavior of the RR of mint samples at different temperatures, which includes three stages: the first stage: the rapid absorption of water in the first minutes; the second stage: reduction in water absorption; and the third stage: constant rate or post-equilibrium from 180 min. These three stages have been observed by other researchers on the RR of Aloe vera [68] and Daucus carota [69]. According to Vega-Galvez et al. [70], the rapid initial absorption of water can be due to the filling of the capillaries on the surface of the sample at the beginning of the process. A decrease in the amount of RR at the end of the process can be associated with a decrease in free capillaries due to the water filling of intracellular spaces [68]. Based on the obtained results, RR was higher in the dried mint samples at higher water temperatures (80 and 90 °C). Rekik et al. [71] stated that increasing the drying rate at higher temperatures causes a more porous structure in the sample and ultimately leads to the creation of more space for water absorption. Of course, it should be noted that increasing the water temperature from 80 to 90 °C caused a decrease in water RR (Table 8).

### 3.8. Antioxidant Activity (AA)

According to Table 5, AA was significantly affected by drying temperature (*p* < 0.01). By examining the effect of temperature on the AA of mint, it was found that drying mint with the RW method reduces the AA compared to fresh samples. The final dried product is the first compared to the fresh product. As is clear from Table 8, the increase in water temperature from 50 °C to 70 °C leads to a slight increase in AA from 58.55 to 71.31%. The reason for this is the creation of Maillard reaction products and/or the release of cellular compounds with antioxidant properties, and applying higher drying temperatures leads to a significant decrease in the AA of the samples (*p* < 0.01). According to López-Vidaña et al. [72], the reduction in AA at higher water temperatures is irreversible due to the oxidation reaction. As can be seen in Table 8, the amount of AA at 80 °C and 90 °C was 73.52 and 67.50%, respectively, and there was a slight downward trend compared to 70 °C water temperature. A similar trend has been observed in the studies of Zalpouri et al. [39] and Uribe et al. [36] for coriander and physalis, respectively.

### 3.9. Phenol and Flavonoid Content

The average values of TPC and TFC compounds are given in Table 8. The average TPC and TFC compounds of fresh mint samples were 48.51 mg GAE/100g DW and 42.57 mg QE/100 g DW. Various factors affect the TPC of mint. The mint variety is one of the most important factors. The results obtained from the experiments and statistical analysis showed that the content of TPC and TFC compounds in mint decreased significantly compared to fresh samples after drying by the RW method (*p* < 0.05—Table 5). A decrease in TPC and TFC after drying compared to fresh samples was observed in the study by Rajoriya et al. [31]. The reduction in TPC compounds due to drying may be caused by their bonding with other compounds such as proteins or by changing the chemical structure of these compounds. On the other hand, the effect of different temperatures on TPC was investigated. According to Table 8, a 30% increase in TPC with increasing temperature from 50 to 70 °C and a 20% decrease in TPC with increasing temperature from 70 to 90 °C was observed. 

According to the report of Harboune et al. [73] and Preethi et al. [35], the reason for the binding of TPC compounds to proteins or changes in their chemical structure, caused by the effect of heat on tannin compounds, is the cellular destruction of the vacuoles inside the phenolic compounds, enzymatic reactions, breaking covalent bonds, and increasing oxidation reactions of bioactive molecules. High temperatures and long drying processes break down tannins, which are hydrolyzable compounds. Therefore, TPC and TFC compounds are sensitive to any change and disappear when faced with temperature [23]. Therefore, the RW drying process at 70 °C tends to retain higher TPC and TFC compounds compared to other process temperatures evaluated. Rakic et al. [74] attributed the observed changes in the TPC compounds at high temperatures to the effect of heat on tannic compounds. According to the research by Uribe et al. [36] on the physalis plant in the RW method, the sample dried at a water temperature of 70 °C has the highest TPC and TFC compounds, and water temperature higher or lower than 70 °C has a negative effect on the phenolic compounds of physalis leaves. Similarly, the results of Nguyen et al. [75] showed that TPC compounds decrease at very high water temperatures.

### 3.10. Essential Oil Efficiency

Based on the results of the analysis of variance, the impact of drying water temperature on the EO was significant (*p* < 0.01—Table 7). The comparison table of the averages showed that the highest EO (2.01 and 1.93%) was obtained at the water temperature of 70 and 80 °C and the lowest value (0.82%) belonged to the water temperature of 50 °C (Table 8-*p* < 0.01). These values in the study by Mokhtarikhah et al. [15] for spearmint (*Mentha spicata* L.) were in the range of 0.2 to 2.3% in different drying methods. Also, the highest and lowest EO for peppermint (*Mentha piperita* L.) were reported as 2.22 and 0.133%, respectively, by Torki-Harchegani et al. [55] in different drying methods. As can be seen in Table 8, increasing the temperature from 40 to 80 °C has increased (148.1%) the efficiency of extracting EO from the dried mint leaves, which can be attributed to the difference in the type of plants, secretion structures of EO maker, and chemical compounds attributed to EO [76]. But with increasing temperature from 80 to 90 °C, the extraction efficiency of EO decreased by 13.9%. In general, the temperature and time required for drying are considered as two important and influential variables on the efficiency of essential oil extraction. The results obtained in this research confirm that low temperatures and a long time during the drying process lead to a decrease in the efficiency of essential oil extraction compared to other temperatures because the long drying time removes a significant part of the volatile compounds that make up the essential oil and leads to a decrease in the extraction efficiency in the dried samples [19]. Torki-Harchegani et al. [55] found that the temperature of 50 °C leads to the production of the lowest amount of EO in peppermint (*Mentha piperita* L.) samples. On the other hand, high temperatures can lead to a decrease in EO due to the acceleration of evaporation, decomposition of EO components, and more damage to the cell wall [15].

### 3.11. Modeling

By calculating the amount of MR for all the studied treatments during the mint drying process (using Equation (1)) and fitting the points obtained from drawing the moisture–time ratio diagrams using the models reported in Table 1, the results for each model were examined. The best model should have the highest value of explanation (R^2^) and the smallest error values (MSE). In Table 9, the R^2^ and MSE are presented. Therefore, the use of the Page model in the RW method is recommended. Asiimwe et al. [77] also recommended using the Page model to investigate the drying kinetics of Passiflora edulis in an RW dryer.

**Figure 5:**
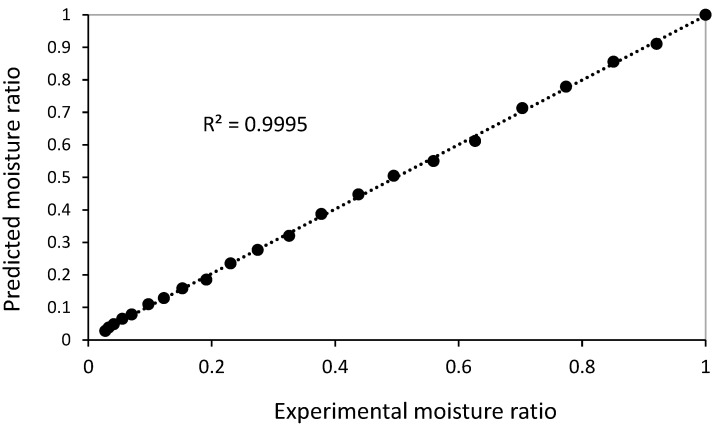
Comparison of experimental and predicted moisture ratio (MR) values of dried mint at 70 °C for Page model.

To check the ability of the proposed Page model, the values of changes in the MR predicted by the Page model and the values of the experimental MR obtained (water temperature 70 °C) are shown in Figure 5. As can be seen in this figure, there is a good agreement between the experimental and predicted MR by the model; therefore, the proposed Page model is suitable for predicting changes in the MR of dried mint samples with RW.

### 3.12. Artificial Neural Network

Artificial neural network is a complex optimization, modeling, and simulation tool that shows high potential due to its powerful prediction and estimation capabilities [78]. In the present study, the nonlinear relationship between two input variables (water temperature and drying time) and an output response (MR) was described through the development of an ANN-based model through the feedforward back propagation algorithm. The network was created using experimental data (MR) including an input layer, one or two hidden layers, and an output layer. The number of neurons in the input and output layers was specified and equal to two (water temperature and drying time) and one (MR), respectively, and as a result, the choice was only limited to determining the appropriate number of neurons in the hidden layer.

In order to choose the most suitable transfer function for the system, various types of activation functions were examined and finally, the Tansig function for the first, Logsig function for the second hidden layer, and Purelin function for the output layer had the best performance compared to the other activation functions. Different configurations (topologies) with different numbers of neurons in the hidden layer were investigated using the design trial and error method and the configurations with the highest R^2^ and the lowest MSE were selected. In this research, the results of the information related to the best activation functions, topology, and MSE and R^2^ values for predicting the moisture ratio by ANN are reported in Table 10.

**Figure 6:**
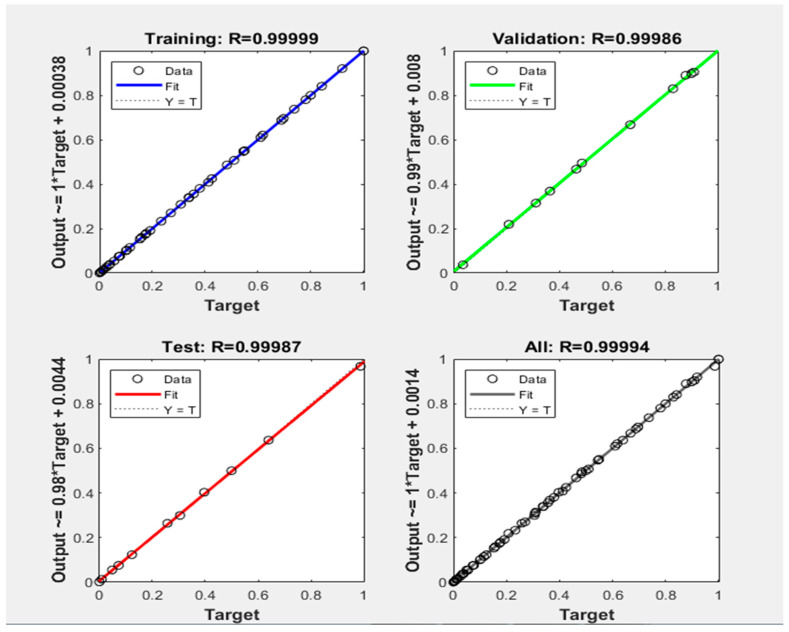
Comparison between the experimental and predicted moisture ratio values during the training, validation, and testing of the best ANN model.

As expected, ANN can successfully predict the moisture ratio of mint drying using the RW method. The predicted values by the ANN model were plotted against the experimental values of both response variables. The fit between the experimental response and prediction by the ANN model is shown in Figure 6. All the points were closer to the straight line, which shows that this model can predict the experimental data for each response variable (MR) with reliable accuracy and validity.

### 3.13. Comparison of Mathematical Models and ANN

In order to compare kinetic models and artificial neural networks, the two criteria of explanation coefficient (R^2^) and mean square error (MSE) were used as the statistical indicators (Table 11). The average value of the coefficient of explanation for the kinetic model and the ANN in predicting the MR was equal to 0.9984–9996 and 0.9999, respectively, which indicates the accuracy and better prediction ability of the ANN model. In addition, the root mean square error values for the kinetic model and the ANN in predicting the MR were determined as 0.0124–0.0178 and 8.77 × 10^−7^, respectively. More deviation between the predicted and actual values (residuals) was observed in the kinetic model compared to the ANN model, and the ANN model showed stable residuals with relatively little changes. The results show that both models provided good predictions, but the artificial neural network has a better prediction capacity compared to the kinetic model, which is related to its overall ability to approximate nonlinear systems. These results showed that compared to the kinetic model, the ANN model is a much better and more accurate predictor as a modeling tool for the nonlinear data of mint moisture ratio. The ANN approach is flexible and allows adding new experimental data to build a more reliable model. Therefore, it will be more logical and reliable to interpret the data of MR of dried mint using the RW method through the ANN architecture process. The predictive superiority of the ANN model over the kinetic model for data fitting and estimation capability has already been shown in previous studies. The results of similar research showed that for predicting the MR of Insulin plant leaves [41] and Annona muricata leaves [40], ANN, due to the higher R^2^ value and lower RMSE, performs better compared to classic models.

## 4. Conclusions

In this research, mint leaves were dried in an RW dryer and the effect of drying water temperature on kinetic parameters, process energy consumption, GHG, thermodynamic parameters, bioactive properties, final product quality, and essential oil efficiency were investigated. According to the obtained results, with the increase in drying water temperature, drying time, energy consumption, CO_2_, and NO_x_ decreased by 63.6%, 50.3%, 48.9%, and 52.1%, respectively, while the rehydration ratio increased from 2.48 × 10^−8^ to 7.28 × 10^−8^ m^2^/s. Enthalpy and entropy values were obtained in the range of 22.76 to 23.10 kJ/mol and −0.1175 to −0.1077, respectively, and they decreased with increasing temperature. The water reabsorption capacity in the dried mint samples was higher at higher water temperatures. Antioxidant activity, total phenol, and flavonoid content in the fresh samples were significantly (*p* < 0.01) higher than in the dry samples. Meanwhile, increasing the drying water temperature from 50 to 70 °C caused a significant (*p* < 0.01) increase in the antioxidant activity, total phenol, and flavonoid content of mint, while increasing higher temperatures caused a significant decrease. The Chroma index and color changes had the opposite trend; that is, with the increase in temperature from 50 to 70 °C, the color changes decreased, while the Chroma index increased. However, with the increase in temperature from 70 to 90 °C, the process of changes was reversed. In general, based on the results obtained in this research, although the use of higher temperatures for drying the samples improved the energy consumption indicators, it decreased the quality of the final product. In this research, the parameter of MR in drying by RW of mint was predicted by different mathematical models and ANN along with different stimulus functions. The comparison of the results of the models obtained from two classical modeling methods and the ANN model showed that the ANN has more power than the classical (mathematical) models in predicting the value of the moisture ratio.

## Figures and Tables

**Table 1:** Mathematical models applied to the drying curves.

Model Name	Model Formula	Reference
Logarithmic	MR=aexp(−kt)+c	[41]
Page	$MR=\exp(-kt^{n})$	[6]
Wang and Singh	$MR=1+at+bt^{2}$	[40]
Henderson and Pabis	MR=aexp(−kt)	[42]
Newton	MR=exp(−kt)	[45]
Two-term	$MR=a\exp(-kt)+c\exp(-k_{0}t)$	[46]

**Table 2:** The results of the variance analysis related to the effect of different drying water temperatures on the drying time, SEC, D_eff_, GHG, and thermodynamic properties.

S.O.V	Time	SEC	D_eff_	CO_2_	NO_x_	ΔH	ΔS	ΔG
df	4	4	4	4	4	4	4	4
Sum Sq	17,802	14.92	4.43 × 10^−15^	6.60	2.41 × 10^−4^	0.20	4 1.78 × 10^−4^	106.4
Mean Sq	4451	3.73	1.10 × 10^−15^	1.65	6.04 × 10^−5^	0.051	4.46 × 10^−5^	26.61
F value	84.72	61.29	77.08	101.6	79.77	44.69	69.96	51.13
Pr (>F)	1.13 × 10^−7^ **	5.36 × 10^−7^ **	1.79 × 10^−7^ **	4.71 × 10^−8^ **	1.52 × 10^−7^ **	2.39 × 10^−6^ **	2.85 × 10^−7^ **	1.27 × 10^−6^ **
C.V	6.94	5.83	8.92	4.39	4.94	0.01	−0.7	1.16

** significant at *p* < 0.01.

**Table 3:** Effect of different water temperatures on the drying time, D_eff_, SEC, CO_2_, and NO_x_ of the dried mint samples during refractance window drying.

T (°C)	Drying Time (min)	D_eff_ (m^2^/s)	SEC (kWh/kg)	CO_2_ (kg/kg Water)	NO_x_ (kg/kg Water)
50	156 ± 4.02 ^a^	2.48 × 10^−8^ ± 9.90 × 10^−10 d^	5.66 ± 0.14 ^a^	3.80 ± 0.08 ^a^	0.023 ± 0.0004 ^a^
60	124.33 ± 4.25 ^b^	2.94 × 10^−8^ ± 1.93 × 10^−9 d^	4.91 ± 0.08 ^b^	3.39 ± 0.04 ^b^	0.020 ± 0.0004 ^b^
70	105.00 ± 2.35 ^c^	3.67 × 10^−8^ ± 1.75 × 10^−9 c^	4.20 ± 0.12 ^c^	2.91 ± 0.04 ^c^	0.017 ± 0.0004 ^c^
80	80.00 ± 2.35 ^d^	4.87 × 10^−8^ ± 2.40 × 10^−9 b^	3.55 ± 0.11 ^d^	2.43 ± 0.07 ^d^	0.014 ± 0.0003 ^d^
90	56.66 ± 3.60 ^e^	7.28 × 10^−8^ ± 2.12 × 10^−9 a^	2.81 ± 0.09 ^e^	1.94 ± 0.03 ^e^	0.011 ± 0.0003 ^e^

Different letters in the columns have a significant difference at the 1% probability level. Similar letters in each column indicate the absence of significant differences based on Duncan’s test at the 5% probability level.

**Table 4:** Effect of different water temperatures on the thermodynamic properties of the dried mint samples during refractance window drying.

T (°C)	ΔH (kJ/mol)	ΔΣ (kJ/mol K)	ΔΓ (kJ/mol)
50	23.12 ± 0.014 ^a^	−0.1077 ± −0.0002 ^a^	57.90 ± 0.37 ^e^
60	23.03 ± 0.079 ^b^	−0.1103 ± 0.0003 ^b^	59.78 ± 0.25 ^d^
70	22.93 ± 0.024 ^c^	−0.1128 ± 0.0004 ^c^	61.67 ± 0.31 ^c^
80	22.89 ± 0.030 ^d^	−0.1152 ± 0.0004 ^d^	63.55 ± 0.23 ^b^
90	22.77 ± 0.017 ^e^	−0.1175 ± 0.0003 ^e^	65.43 ± 0.47 ^a^

Different letters in the columns have a significant difference at the 1% probability level.

**Table 5:** The results of the variance analysis related to the effect of different drying water temperatures on some color and bioactive properties.

S.O.V	L*	a*	b*	TPC	TFC	AA
df	5	5	5	5	5	5
Sum Sq	175.63	16.29	94.21	610.4	522.6	1720.4
Mean Sq	35.13	3.25	18.84	122.09	104.5	344.1
F value	28.23	7.53	68.93	6.05	5.17	15.8
Pr (>F)	3.05 × 10^−6^ **	0.002 **	2.04 × 10^−8^ **	0.005 **	0.009 **	6.44 × 10^−5^ **
C.V	2.97	−7.66	2.12	10.74	13.39	6.52

** significant at *p* < 0.01.

**Table 6:** Effect of different water temperatures on the chromatic properties of the dried mint samples during refractance window drying.

T (°C)	L*	a*	b*	ΔE	C
Fresh	42.89 ± 0.86 ^a^	−10.80 ± 0.73 ^d^	27.87 ± 0.69 ^a^	-	29.65 ± 0.83 ^a^
50	32.75 ± 0.47 ^e^	−7.18 ± 0.23 ^a^	21.28 ± 0.09 ^e^	13.00 ± 0.03 ^a^	22.46 ± 0.16 ^e^
60	35.46 ± 0.56 ^d^	−8.50 ± 0.24 ^bc^	23.35 ± 0.09 ^d^	11.56 ± 0.04 ^b^	24.85 ± 0.17 ^d^
70	39.03 ± 0.47 ^b^	−9.13 ± 0.17 ^cd^	26.93 ± 0.09 ^b^	9.96 ± 0.07 ^d^	28.43 ± 0.14 ^b^
80	37.77 ± 0.41 ^bc^	−8.72 ± 0.19 ^bc^	25.32 ± 0.09 ^c^	10.23 ± 0.04 ^d^	26.78 ± 0.15 ^c^
90	36.90 ± 0.51^cd^	−7.77 ± 0.20 ^ab^	23.12 ± 0.09 ^d^	10.78 ± 0.01 ^c^	24.39 ± 0.15 ^d^

Different letters in the columns have a significant difference at the 1% probability level.

**Table 7:** The results of the variance analysis related to the effect of different drying water temperatures on the rehydration ratio, color, Chroma, and essential oil.

S.O.V	RR	ΔE	Chroma	EO
df	4	4	4	4
Sum Sq	2.59	17.89	63.21	3.08
Mean Sq	0.64	4.47	15.80	0.77
F value	21.64	91.81	141.6	74.28
Pr (>F)	6.54 × 10^−5^ **	7.68 × 10^−8^ **	9.28 × 10^−9^ **	2.14 × 10^−7^ **
C.V	3.88	1.98	1.31	6.59

** significant at *p* < 0.01.

**Table 8:** Effect of different water temperatures on the bioactive properties and essential oil of the dried mint samples during refractance window drying.

T (°C)	RR	TPC (mg GAE/100g DW)	TFC (mg QE/100 g DW)	AA (%)	EO (%)
Fresh	-	48.51 ± 4.90 ^a^	42.57 ± 2.95 ^a^	89.61 ± 4.95 ^a^	-
50	3.79 ± 0.06 ^d^	31.18 ± 0.70 ^c^	26.52 ± 0.46 ^c^	58.55 ± 0.90 ^d^	0.82 ± 0.03 ^d^
60	4.25 ± 0.08 ^c^	40.72 ± 0.86 ^ab^	28.62 ± 0.61 ^bc^	64.92 ± 0.82 ^cd^	1.23 ± 0.04 ^c^
70	4.50 ± 0.05 ^bc^	46.42 ± 0.40 ^ab^	36.81 ± 0.82 ^ab^	75.31 ± 1.41 ^b^	1.93 ± 0.07 ^a^
80	5.01 ± 0.10 ^a^	45.19 ± 0.66 ^ab^	35.47 ± 0.71 ^ab^	73.52 ± 0.73 ^bc^	2.01 ± 0.04 ^a^
90	4.72 ± 0.08 ^ab^	38.52 ± 0.70 ^bc^	31.34 ± 0.78 ^bc^	67.50 ± 0.70 ^bc^	1.73 ± 0.01 ^b^

RR: rehydration ratio, TPC: total phenol content, TFC: total flavonoid content, and EO: essential oil. Different letters in the columns have a significant difference at the 1% probability level

**Table 9:** Statistical analysis reports of different mathematical models for mint leaves in RW dryer.

Model	T (C)	R^2^	MSE
Logarithmic	50	0.9958	0.0302
	60	0.9972	0.0263
	70	0.9944	0.0315
	80	0.9969	0.0244
	90	0.9981	0.0199
	Average	0.9964	0.0264
Page	50	0.9996	0.0124
	60	0.9990	0.0148
	70	0.9995	0.0130
	80	0.9988	0.0169
	90	0.9984	0.0178
	Average	0.9990	0.0149
Newton	50	0.9696	0.0710
	60	0.9858	0.0515
	70	0.9803	0.0595
	80	0.9900	0.0415
	90	0.9729	0.0677
	Average	0.9797	0.0582
Wang and Singh	50	0.9965	0.0268
	60	0.9908	0.0411
	70	0.9952	0.0299
	80	0.9798	0.0622
	90	0.9868	0.0496
	Average	0.9898	0.0419
Two-term	50	0.9867	0.0488
	60	0.9893	0.0424
	70	0.9811	0.0611
	80	0.9659	0.0722
	90	0.9903	0.0418
	Average	0.9826	0.0532
Henderson and Pabis	50	0.9985	0.0173
	60	0.9968	0.0245
	70	0.9970	0.0242
	80	0.9860	0.0512
	90	0.9862	0.0511
	Average	0.9929	0.0336

**Table 10:** Best selected topologies including training algorithm, different layers, and neurons for ANN to predict moisture ratio.

Threshold Function	Number of Layers and Neurons	MSE	R^2^	Epoch
Tansig-Tansig-Tansig	2-10-10-1	0.000312	0.9975	12
Tansig-Purelin-Tansig	2-10-10-1	1.73 × 10^−6^	0.9999	147
Tansig-Purelin,	2-10-1	2.45 × 10^−6^	0.9998	120
Tansig-Logsig-purelin,	2-15-14-1	8.77 × 10^−7^	0.9999	15
Tansig-Tansig	2-15-1	0.0041	0.9828	15

**Table 11:** Comparative study of ANN and the best semi-empirical model (page model).

Model	MSE	R^2^
ANN	8.77 × 10^−7^	0.9999
Page	0.0124–0.0178	0.9984–9996

## Data Availability

The original contributions presented in the study are included in the article, further inquiries can be directed to the corresponding author.

## Nomenclature

AA	%	Antioxidant activity
A_Sample_	-	Absorbance values of the blank
A_control_	-	Absorbance values of the sample
D_eff_	m^2^/s	Effective moisture diffusivity
E_a_	J/mol	Activation energy
h_p_	6.626 × 10^−37^ m²·kg/s	Planck constant
K_b_	1.38 × 10^−26^ J/K	Boltzmann constant
L	m	The thickness of the sliced cantaloupe
M_e_	% d.b	Equilibrium moisture contents
MR	-	Moisture ratio
M_o_	% d.b	Final
		The moisture content of the sample
M_t_	% d.b	The moisture content at time t
${MR}_{exp,i}$	-	Experimental moisture fraction
${MR}_{pre,i}$	-	Predicted moisture fraction
N	-	Number of observations.
R	8.314 J/mol. K	Universal gas constant
RR	-	Rehydration ratio
W_r_	g	Weight of the rehydrated sample (g)
W_d_	g	Weight of the dry sample (g)
SEC	kWh/kg	Specific energy consumption
t	s	Drying time
T	K	Absolute temperature
∆H	J/mol	enthalpy
∆G	J/mol	Gibbs free energy
∆S	J/mol. K	Entropy
∆L*, ∆b*, ∆a*	-	The difference between the color of the fresh and dried samples
